# Cervical cancer in Cape Verde: reappraisal upon referral to a tertiary cancer centre

**DOI:** 10.3332/ecancer.2022.1471

**Published:** 2022-11-17

**Authors:** Ana Teresa Vilares, Riccardo Ciabattoni, Teresa Margarida Cunha, Ana Félix

**Affiliations:** 1 Department of Radiology, Centro Hospitalar Universitário de São João, Porto 4200-319, Portugal; 2 Medical School, University of Porto, Porto 4200-319, Portugal; 3 Azienda Sanitaria Universitaria Giuliano-Isontina, Dipartimento Universitario di Scienze Mediche Chirurgiche e Sperimentali, Università degli Studi di Trieste, Trieste 34148, Italy; 4 Department of Radiology, Instituto Português de Oncologia de Lisboa Francisco Gentil, Lisbon 1099-023, Portugal; 5 Department of Pathology, Instituto Português de Oncologia de Lisboa Francisco Gentil, Lisbon 1099-023, Portugal; 6 NOVA Medical School, NMS, Universidade NOVA de Lisboa, Lisbon, Portugal; a https://orcid.org/0000-0001-7375-491X; b https://orcid.org/0000-0003-4555-6128; c https://orcid.org/0000-0003-2411-0207; d https://orcid.org/0000-0002-2653-2262

**Keywords:** cervical cancer, Cape Verde, HPV human papillomavirus, risk factors, epidemiology

## Abstract

**Background:**

Cervical cancer (CC) is the first cause of cancer-related deaths among Cape Verdean women. The absence of a national screening programme and a lack of dedicated cancer treatment facilities contribute to its high mortality rate. In an effort to improve the prognosis of these women, a health cooperation agreement was established between Portugal and Cape Verde (CV), allowing their evacuation to Portuguese hospitals. Our aim was to characterise CC among CV women, and to assess the response given to these patients in Instituto Português de Oncologia de Lisboa Francisco Gentil (IPOLFG), so that their treatment and follow-up protocols can be optimised and overall prognosis improved.

**Methods:**

Retrospective evaluation of women diagnosed with CC in CV that underwent therapy in IPOLFG between 2013 and 2020. Risk factors, demographic and tumour characteristics, treatment and outcomes were reviewed.

**Results:**

Fifty-eight patients were included. Squamous cell carcinoma was the most frequent (91.5%) histological type. HPV DNA was present in 25 out of 26 samples.The agreement rate between the pathology analysis performed in CV and in Portugal was high (87.9%); however, the agreement regarding the FIGO stage was low (15.5%). This may be explained by both the time interval between diagnosis and treatment (around 6 months) and by the absence of resources to accurately stage the disease in CV. In IPOLFG, 77.6% of patients received combined chemo-radiotherapy. Post-treatment follow-up varied widely, due to disease-related and bureaucratic issues. Eighteen patients developed cancer-related complications and/or cancer-related death. The survival rate and median overall survival (OS) in our cohort were of 89.7% and 73.2 months, respectively.

**Conclusions:**

Although most women had advanced-stage disease, the OS in our cohort was better than what has been reported for other African countries, probably because state-of-the-art treatment, frequently not accessible in those countries, was offered to all patients.

## Introduction and background

Cervical cancer (CC) is the fourth most frequently diagnosed cancer among females worldwide, with an estimated 604,000 new cases and 342,000 deaths reported in 2020 [1]. The global burden of CC is unevenly distributed, with approximately 90% of all the new cases and deaths occurring in low- and middle-income countries (LMICs) in 2020 [1]. Sub-Saharan Africa (SSA) is the region with the highest incidence and mortality rate of CC, and this is the most frequently diagnosed cancer in females in half of the SSA countries [2]. Cape Verde (CV) is a sub-Saharan western African country that belongs to the community of Portuguese-speaking African countries (*Países Africanos de Língua Oficial Portuguesa* – PALOP), which also includes Angola, Guinea-Bissau, Mozambique, São Tomé and Príncipe and Equatorial Guinea. CV has an estimated population of 196,716 women aged 15 years and older, who are at risk of developing CC. In CV, CC is the third most frequently diagnosed cancer among women between 15 and 49 years of age, with an estimated incidence of 17 per 100,000 women, which is only surpassed by thyroid and breast cancers [3]. More importantly, CC is the first cause of cancer-related death in females in CV, causing 10.5 deaths per 100,000 women. Overall, Cape Verdean women have a cumulative risk of developing CC (between 0 and 74 years of age) of 1.8%, and a cumulative risk of CC-related death of 1.23% [3]. The high incidence and mortality rates of CC in CV may be partly explained by both the absence of a national screening programme and by the fact that the human papillomavirus (HPV) vaccination programme was only initiated in 2021 [4]. Similar to what happens in many other SSA countries, cytology-based screening has not yet been implemented, given the lack of adequate health infrastructure, resources and expertise [5]. When opportunistic screening is performed, the most frequently used test is visual inspection with acetic acid, whose sensitivity and specificity are lower than cytology-based screening [6]. Moreover, in these countries, the great majority of women only seek healthcare after experiencing cancer-related symptoms, such as vaginal bleeding, abnormal vaginal discharge or dyspareunia. Since symptomatic presentation occurs late in the disease course, typically when it has reached an invasive ulcerating stage, this results in a worsened overall prognosis and increased mortality rates [5–7]. It should also be noted that little is known about the demographics and risk factors for developing CC among Cape Verdean women. Most importantly, there are no data regarding the prevalence and genotyping of HPV in CV [3], even though infection with high-risk types (including HPV 16 and 18) is known to be implicated in the development of nearly all CC [8]. Data regarding other risk factors for the development of CC, such as smoking, parity, age of first sexual intercourse, number of sexual partners and human immunodeficiency virus (HIV) infection are also absent or scarce [3, 5].

The treatment of CC in CV is highly limited by the absence of adequate health infrastructures and the lack of human and physical resources. There are no dedicated cancer treatment centres neither tertiary care cancer departments and, until 2020, national guidelines for CC management had yet not been implemented [4]. Chemotherapy is available but dedicated oncologic surgery and radiotherapy departments do not exist [4]. All the above-mentioned factors contribute to the high incidence and mortality rates of CC reported in CV. In an effort to overcome these difficulties, a health cooperation agreement has been established between Portugal and CV since 1976 [9]. This cooperation programme allows up to 300 Cape Verdean patients per year to be evacuated to Portugal to proceed with complex medical and/or surgical treatments [10]. Within the scope of this agreement, Cape Verdean women diagnosed with CC are frequently evacuated to Instituto Português de Oncologia de Lisboa Francisco Gentil (IPOLFG), a tertiary cancer centre located in Lisbon. This cooperation agreement is of the utmost importance, since it allows Cape Verdean women to be offered state-of-the-art treatment for CC, that is not available in their country of origin. Through this retrospective study, we aim to emphasise the relevance of this cooperation programme in the prognosis and overall survival (OS) of Cape Verdean women diagnosed with CC, and to monitor the Portuguese health service response to these patients. The outcome of the patients, including evaluation of disease persistence, recurrence, metastatic spread and/or cancer-related death will be evaluated and compared to the outcomes described in the literature for other SSA countries. With this study, we intend to highlight the disparities in access to quality healthcare between LMICs and high-income countries, and to emphasise how health cooperation agreements between those countries can lead to improved patient outcomes.

## Methods

### Study design

We conducted a descriptive retrospective single-centre study. We reviewed all cases of women diagnosed with CC in CV and treated in IPOLFG in the period between January 2013 and December 2020, within the scope of the health cooperation agreement established between CV and Portugal. The referral to IPOLFG was determined by the local Cape Verdean clinical teams, based on the need for specialised cancer care that was not available in CV (e.g. need of radiotherapy treatments or dedicated oncological surgery). All patients referred for transferral were accepted by IPOLFG. Using the local clinical system of IPOLFG, we identified 70 patients that fulfilled the inclusion criteria. Patients were then excluded from the study if the initial diagnosis of CC was not made in CV (e.g. Cape Verdean women living in Portugal; *n* = 10), and if the final pathological diagnosis was not CC (e.g. disagreement between the diagnosis in CV and in IPOLFG; *n* = 2). These exclusions resulted in a total of 58 patients with histologically confirmed CC diagnosed in CV and treated in IPOLFG. The study was approved by the local ethical and scientific committees (nº UIC1372).

### Eligibility and assessment of relevant clinical data

Physical and electronic clinical records of the selected patients were reviewed. Relevant biographic, demographic and risk factor data were collected, and included: age at diagnosis of CC, age at menarche, age at menopause (if applicable), number of pregnancies, number and type of births, age at first sexual intercourse (AFSI), number of sexual partners, familiar history of gynaecologic or breast cancer, tobacco smoking, immunosuppression status, HIV and HPV status, as well as HPV genotype (when applicable).

Data regarding the tumour characteristics were also collected, including the histological type and FIGO staging determined in CV and the histological type and FIGO staging determined in IPOLFG after multidisciplinary team evaluation. The agreement rate between the histological diagnosis and FIGO staging attributed in CV and in IPOLFG was then calculated.

Information regarding the type of radiological examinations performed for tumour staging in CV and in IPOLFG was also evaluated. Tumour characteristics relevant for staging [11] were assessed in the available radiological exams.

Data about the type and date of treatments performed prior to admission at IPOLFG, and regarding the type and date of medical and/or surgical treatments performed at IPOLFG, were also collected. When applicable, the time interval between the initial diagnosis of CC in CV and the beginning of treatments in IPOLFG was calculated.

### Radiological exams

Patients underwent magnetic resonance imaging (MRI) in IPOLFG, either in a 1.5T or 3T MR scanner, using the protocol established by the European Society of Urogenital Radiology Guidelines for Cervical Cancer staging [12]. All images were reviewed by three radiologists with, respectively, 4 years, 5 years and 25 years of female pelvis imaging experience.

When appropriate, and according to current guidelines for CC management [13], CT and/or positron emission tomography (PET)-CT was also performed in our institution.

Based on the clinical assessment, radiological and nuclear medicine exams, a FIGO stage was assigned to each tumour. Disagreements were discussed in the gynaecological multidisciplinary team meeting, held weekly, in order to attain a definitive FIGO staging.

### Pathological exams

Histological confirmation of CC diagnosis was always performed locally, before treatment decision. All relevant gross (pathological report) and microscopic examinations (biopsy or surgery) performed in CV were sent from local pathology departments to the IPOLFG Pathology Department for revision. p16 immunohistochemistry was performed in all cases diagnosed after 2020, according to the revised WHO guidelines [14]. Before 2020, p16 immunohistochemistry was only performed if requested by the clinicians. Histological results were collected from physical and electronic records and, whenever possible, the agreement rate between the pathological exams performed in CV and in IPOLFG was assessed.

### HPV detection and typing

HPV DNA detection was performed from tumour biopsies collected in Thin Prep conservative medium (ThinPrep Pap Test Hologic*^→^*) [15]. The DNA was extracted using a spin silica filter column. The real-time PCR (qPCR) was performed by SYBR GREEN dye to amplify a 65-bp region within the L1 region consensus primers (SPF10) [16]. The internal amplification control (albumin gene) was performed in independent reactions along with HPV detection. A melting curve was performed to confirm the specificity (Tm 73°C) of the 75pb amplicon. The genotyping of the positive samples was processed by commercial kit, LiRas assay (INNO-LiPA^®^ HPV Genotyping Extra II | Fujirebio) that detects 32 different HPV types, including 13 hrHPV (HPV16, HPV18, HPV31, HPV33, HPV35, HPV39, HPV45, HPV51, HPV52, HPV56, HPV58, HPV59 and HPV68), 6 possible hrHPV (HPV26, HPV53, HPV66, HPV70, HPV73 and HPV82), 9 low-risk HPV (HPV6, HPV11, HPV40, HPV42, HPV43, HPV44, HPV54, HPV61 and HPV81) plus 4 other HPV genotypes (HPV62, HPV67, HPV83 and HPV89) [17].

### Treatment and outcome

Physical and electronic records were reviewed for information regarding medical and surgical treatments performed for each patient. Data regarding chemotherapy (including the number of cycles and type of chemotherapeutic drugs used), radiotherapy and/or brachytherapy (BT) (including the number of sessions and duration of treatments) were collected. The type and date of surgical treatments performed were also assessed.

Finally, we assessed the duration of post-treatment follow-up and evaluated the disease response to treatments and/or the pattern of disease progression. Unfavourable disease course was considered if tumour persistence, recurrence, metastatic spread or cancer-related death occurred.

### Statistical analysis

Categorical variables are presented as frequencies and percentages. Continuous variables were evaluated for normal distribution using skewness and kurtosis. Continuous variables with normal distribution are presented as means and standard deviations, and variables with non-normal distribution as medians and interquartile ranges. OS curve was calculated using the Kaplan–Meier method and the log-rank test was used to assess survival differences between groups. OS was defined from the date of histological diagnosis of the disease to the date of death from any cause or last follow-up. Data were stored and all statistical analyses were performed using the Statistical Package for Social Sciences (SPSS, IBM Corp*^→^*, Chicago, IL, USA) software, version 26.0.

## Results

### Sample demographics and risk factors

Fifty-eight patients met the inclusion criteria and were included in the study. The median age at diagnosis of CC was 56.0 years. The median age at menarche was 13.5 years. Thirty-one females were in menopause upon CC diagnosis, with a median age at menopause of 48 years.

The median number of pregnancies and births were both of 6.5, and none of the females was nulliparous. The vast majority of women had vaginal deliveries, with only three caesareans reported in three different patients. Of the 58 females included, 22 had at least one previous abortion.

Regarding other known risk factors for CC development, information about AFSI was only available for 23 out of the 53 females included (43%). Within this subgroup, 20 females (87%) had less than 18 years at their first sexual intercourse, with a median AFSI of 15.5 years. Information regarding the number of sexual partners was available for 34 of the 58 patients included (59%), with a median number of sexual partners of 1.5.

Data about smoking habits were available for 41 women (71%), with only 2 active smokers (3.4%) and 1 ex-smoker (1.7%).

Regarding immunosuppression status, information was available for all the included patients. Seventeen women (29.3%) were immunosuppressed, out of which 14 had untreated hepatitis B virus (HBV) infection, 1 had untreated hepatitis C virus (HCV) infection and 2 had untreated HIV infection.

Data regarding family history of cancer were available for 33 women (57%), out of which only 2 patients (3.4%) had positive family history of gynaecologic or breast cancer.

Information regarding HPV status was available in 26 women (45%). Of those, 25 females (96%) had confirmed HPV infection. The majority of the infections were caused by HPV 16. Tables 1 and 2 summarise the most relevant demographic and risk factors of our cohort. Table 3 details the HPV genotyping profile.

### Imaging and pathology – findings and agreement

Regarding radiological exams performed for CC diagnosis and staging, our results reveal that, for the vast majority of patients, no exams were performed prior to evacuation to Portugal. In fact, MRI and PET are not available in CV and although CT is available, it was only performed in four women (6.9%).

As such, after evacuation to Portugal, 57 women (57/58; 98.3%) underwent MRI in IPOLFG. Due to clinical contra-indications, one patient could not perform MRI, and an abdomino-pelvic CT was obtained instead. Apart from the case explained, seven other women underwent CT in IPOLFG, to complete staging, according to current CC management guidelines [13]. PET-CT was also performed, when appropriate, according to current guidelines [13]. Thirty patients performed a PET-CT and twenty-nine of those exams (94%) revealed positive pathologic findings. Cervical tumour dimensions were assessed for all cases, and the median greatest axis of the tumours was of 5.4 cm (3.6–7.2 cm).

**Figure 1.**
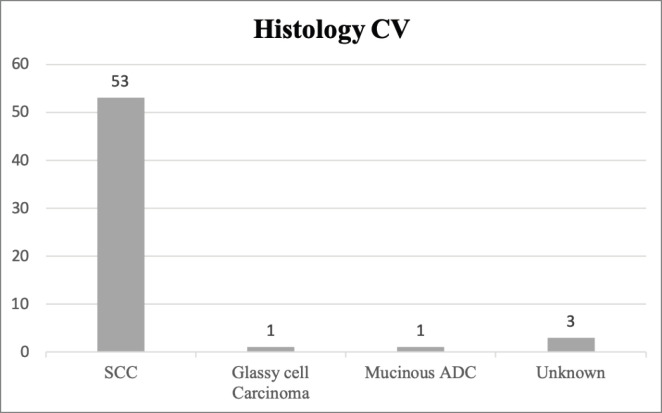
Histology classification of CC in CV. The absolute number of cases of each histological type of CC is presented. SCC, Squamous cell carcinoma; ADC, Adenocarcinoma.

Regarding histology, 53 tumours were classified as squamous cell carcinoma (SCC) in CV, corresponding to 91.4% of our sample. The pathology results from CV are summarised in Figure 1.

**Figure 2.**
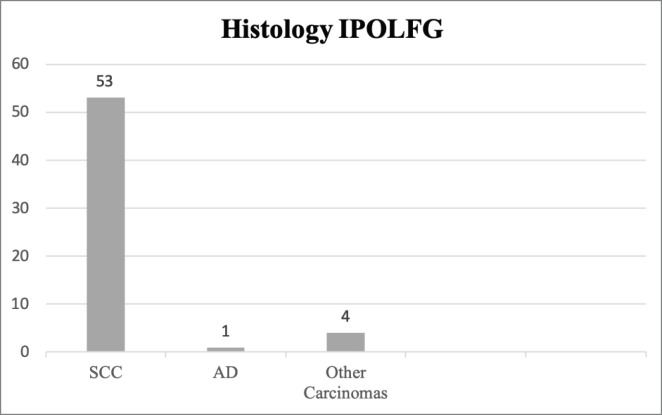
Histology classification of CC in IPOLFG. The absolute number of cases within each histological type of CC is presented. SCC, Squamous cell carcinoma; AD, Adenocarcinoma of the usual type.

In IPOLFG, all cases were reviewed by experienced pathologists in the field of gynaecological malignancies. Fifty-three cases (91.4%) were confirmed as SCC, one (1.7%) as adenocarcinoma and four (6.9%) as other types of carcinoma (including adenosquamous and undifferentiated carcinoma). The histology results from IPOLFG are summarised in Figure 2.

**Figure 3.**
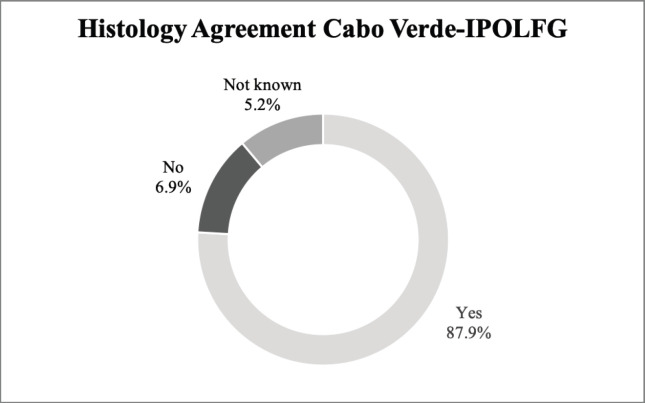
Agreement between the histological exams performed in CV and in IPOLFG. The percentages of agreement (yes) and disagreement (no) between the histology attributed in CV and IPOLFG are presented. The percentage of cases where it was not possible to assess agreement (not known) is also presented.

Our results reveal an 87.9% agreement rate between the pathology analysis performed in CV and the revision performed in IPOLFG. Figure 3 summarises the histological agreement between CV and IPOLFG pathology analysis.

**Figure 4.**
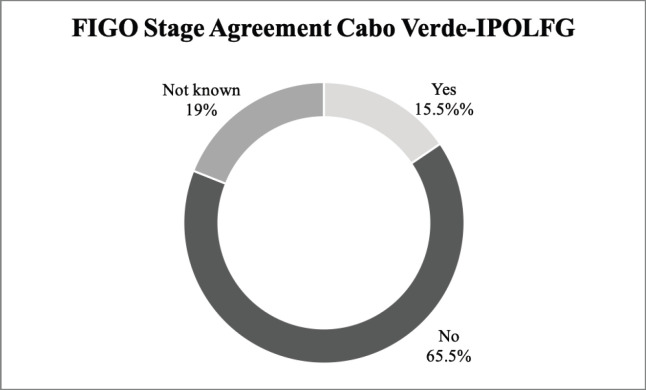
Agreement between the FIGO stage attributed in CV and in IPOLFG. The percentages of agreement (yes) and disagreement (no) between the between the FIGO stage attributed in CV and IPOLFG are presented. The percentage of cases where it was not possible to assess agreement (not known) is also presented.

On the other hand, our results reveal a very low agreement rate (15.5%) between the FIGO staging attributed in CV and the final FIGO staging attributed in IPOLFG. In 65.5% of the cases, there was disagreement regarding the FIGO stage and for 11 patients (19%) it was not possible to evaluate agreement since information about FIGO staging in CV was unavailable. The agreement between the FIGO stage attributed in CV and in IPOLFG is summarised in Figure 4.

Table 4 summarises the FIGO stages attributed in CV and the final FIGO stages attributed in IPOLFG. It should be highlighted that the majority (53.5%) of the women had advanced stage disease (FIGO stage III or IV) in IPOLFG, whereas in CV only 15.5% of patients were classified as having stage III disease and 0% had stage IV disease.

### Treatment and results

In CV, ten women (17%) received chemotherapy and two women (3%) had surgery. Radiotherapy is not available in CV, and therefore it was not performed before evacuation for Portugal.

For the 46 patients who did not receive any treatment in CV (79%), the median time interval between the initial diagnosis and the start of treatment in IPOLFG was 6 months.

In IPOLFG, most patients (*n* = 45; 77.6% of our cohort) received combined chemo-radiotherapy. The standard treatment consisted of six cycles of platinum-based chemotherapy and external RT. Twenty-nine of these patients also underwent brachytherapy.

Surgery was performed in nine patients (15.5%), and for two patients it was the only treatment performed, while for the remaining seven, post-surgical adjuvant treatment was applied. Three patients (5.2%) were treated with combined external RT and BT, and one patient (1.7%) was treated with isolated platinum-based chemotherapy.

All patients had their treatment strategy delineated in a multidisciplinary setting, according to the guidelines for CC treatment [13, 18].

Post-treatment follow-up varied widely among the females included in our study. The main reason for this variation in the duration and timing of follow-up is due to disease-related issues, in addition to bureaucratic, personal and familial issues since the patients were dislocated from their country of origin. For clarity, we divide our study population into three subgroups, according to their post-treatment follow-up.

The first subgroup corresponds to women that were considered to be successfully treated in IPOLFG and is composed by 31 women (53% of our cohort). Five of these women had stage I disease (16.1%), 12 had stage II disease (38.7%), 11 had stage III disease (35.5%) and 3 had stage IV disease (9.7%). None of those women developed disease persistence, recurrence or metastatic spread and all were considered to be disease-free at the end of follow-up. The median duration of follow-up within this subgroup of patients was only 6 months [5–9]. All these patients returned to CV with adequate and personalised follow-up recommendations. It was not possible to retrieve any further information from CV about this subset of patients.

The second subgroup also includes successfully treated women, but that are still being followed-up routinely on IPOLFG, due to their personal choice not to return to CV. This subgroup is composed by nine women (16% of our cohort), four of them with stage I disease (44.4%) and five with stage III disease (55.6%). As expected, the duration of follow-up in this subgroup was much higher (median: 54 months; (30–72 months)). None of the women included in this subgroup developed disease-related complications.

The third subgroup includes all the patients that developed cancer-related complications – persistence, recurrence, metastatic spread and/or cancer-related death. This subgroup includes 18 women (31% of our cohort), out of which 2 (11.1%) had stage I disease, 4 (22.2%) had stage II disease, 8 (44.4%) had stage III disease and 4 (22.2%) had stage IV disease at diagnosis. The median duration of follow-up in this subgroup was 32 months (10–48 months).

Within this subgroup, we documented ten cases of disease persistence (17.2%), two cases of disease recurrence (3.4%) and seven cases of distant metastatic spread (12%). The majority of patients had multifocal metastasis, with the most commonly affected sites being the lungs (*n* = 5), liver (*n* = 3) and peritoneum (*n* = 3). Skeletal metastasis was only encountered in one patient.

Six cancer-related deaths were documented (10.3%), resulting in an OS rate of 89.7%. The number of deaths per FIGO stage was of one in stage II, two in stage III and three in stage IV. The median OS time for the deceased patients was of only 13 months (11–26 months).

**Figure 5.**
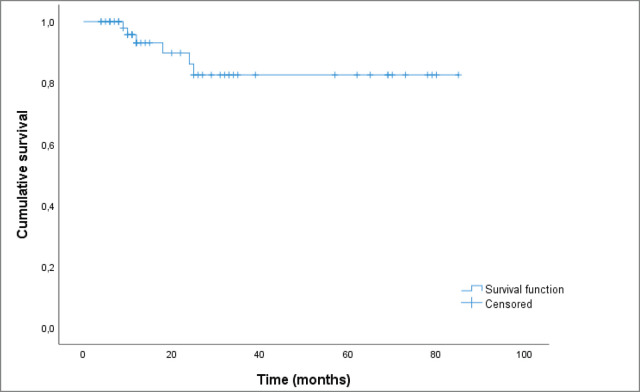
Kaplan–Meier curve showing OS.

Based on the Kaplan–Meyer analysis (Figure 5), the median OS in our cohort was of 73.2 months.

**Figure 6.**
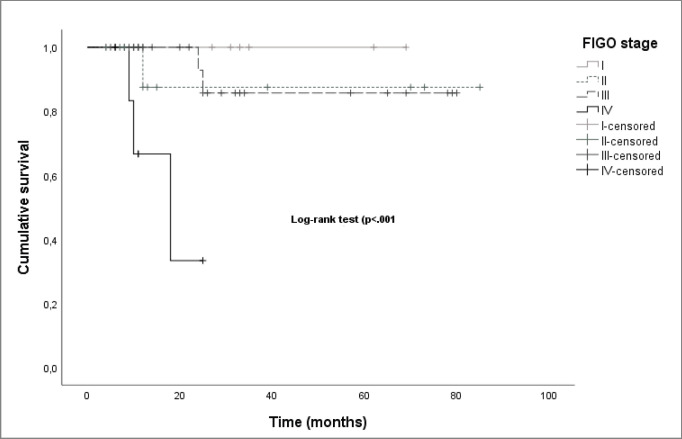
Kaplan–Meier curves by FIGO stage.

The median OS was significantly different between stages (27.0 months for stage I, 11.5 months for stage II, 25.0 months for stage III and 11.1 for stage IV, log-rank-*p* < 0.001) (Figure 6).

**Figure 7.**
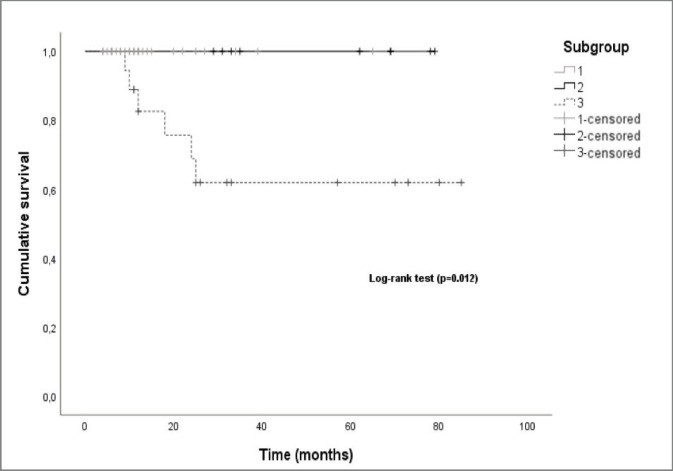
Kaplan–Meier curves by patient subgroup.

Regarding the subgroup of patients, the median OS was also significantly different (log-rank-*p* = 0.012) between the subgroups, with subgroup 2 and subgroup 3 having a median OS of 62 and 25 months, respectively (Figure 7).

However, it should be noted that the median follow-up time for all the patients included in our cohort was of only 14.5 months (10.0–33.3).

## Discussion

### Risk factors

Risk factors for CC development have been extensively described in the literature [7, 19–21], with the main one being HPV infection [22]. Persistent infection with high-oncogenic risk HPV types (HR-HPV) is considered a necessary, although not sufficient, cause for CC development [23]. In fact, HPV DNA has been shown to be present in up to 99.7% of cervical tumour specimens [22]. However, in the vast majority of SSA countries, information regarding the prevalence of HPV infection is scarce [8]. CV is a paramount example of that, since there is no national HPV screening programme in place, and therefore there are no official data regarding the prevalence and genotyping of HPV infection in CV [3, 4]. In our cohort, 25 out of 26 females (96%) had confirmed HPV infection. Our results are in line with data published in the literature about HPV positivity rates among women with CC in other Western African countries, which range from 64.7% in Senegal to 96.9% in Mali [24]. The most common HPV type in our sample was HPV16 (*n* = 18), followed by HPV33, 53 and 68. Several studies have reported HPV16 to be the most commonly detected HPV type in SSA women diagnosed with CC [24–26], which is in accordance with our results. However, literature [24–26] reports that the second and third most frequently detected HPV types in SSA countries are HPV18 and HPV45, which were rarely encountered in our study (one case of each type) and this might be related to the very low number of adenocarcinomas present in our series.

Seventeen of the women included (29.3%) were immunosuppressed at the time of CC diagnosis, out of which 14 had untreated HBV infection, 1 had untreated HCV infection and 2 had untreated HIV infection. In our cohort, we found a higher prevalence of HIV (*n* = 4; 6.8%) and HBV infections (*n* = 14; 24.1%), than what is reported for the general CV population [27, 28], probably due to the fact that HIV infected women have a higher risk of HR-HPV infection [29], and also that immunocompromised women in general have a significantly increased risk of developing CC [30, 31].

Regarding other known risk factors for CC development, including number of pregnancies, AFSI, number of sexual partners and tobacco smoking, our results are in accordance to those described in the literature for other SSA countries [19, 21, 28, 32–37].

### Diagnosis

For all the women included in the study, CC diagnosis was established in CV, after pathological examination of biopsy or surgical specimens, and then confirmed by the Pathology Department of IPOLFG. The most frequent histologic type diagnosed in both CV and IPOLFG was SCC, which is in accordance with published literature [7]. It should be emphasised that there was high agreement (87.9%) between the histological diagnosis established in CV and IPOLFG, as expected for a H&E based diagnosis done by trained pathologists.

On the contrary, there were significant discrepancies between the FIGO staging attributed in CV and the final FIGO staging attributed in IPOLFG, and several factors may have contributed to the observed differences.

First of all, in 19% of our cohort, there was no information regarding the FIGO stage at diagnosis in CV. Since our sample includes patients diagnosed with CC as early as January 2013, patients were staged according to either 2009 [38] or 2018 [11] FIGO guidelines. 2009 guidelines mandated that CC staging should be done surgically or, when surgery was not possible or available, clinically. Since then, it has been shown that clinical staging leads to major inaccuracies [39, 40]; therefore, FIGO guidelines were revised in 2018 [11]. This revision led to the inclusion of both imaging and pathology examinations, whenever possible, in order to supplement clinical findings and increase staging accuracy. Therefore, the absence of FIGO staging in 19% of our cohort highlights the limited access to quality healthcare in CV, a reality most similar to other SSA countries.

**Figure 8.**
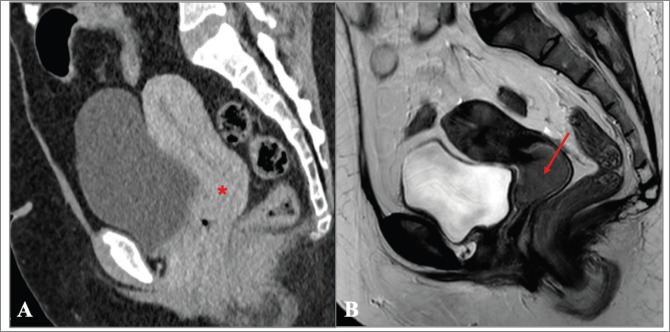
(a and b): Comparison between the CT performed in CV and MRI obtained in IPOLFG. (a): On the sagittal plane CT of the pelvis obtained in the venous phase, the cervix appeared normal (*) and no cervical tumour could be accurately depicted. (b): On the T2-weighted MR image in the sagittal plane performed 3 months later, the cervical tumour was clearly seen, measuring 3.5 cm of greatest axis (FIGO stage IB2).

In the remaining 81% of women FIGO staging was performed, but the agreement with the final staging attributed in IPOLFG was extremely low (15.5%). There are several possible explanations for the major discrepancies encountered. One is related to the limited access to quality healthcare in CV. MRI has been shown to be the ideal method for locoregional staging of CC [12, 41, 42], however it is not available in a significant proportion of SSA countries, of which CV is an example [3, 4]. Although CT exists in CV, it is not easily accessible for the vast majority of the population and it is not as accurate as MRI for locoregional staging of CC [41, 42]. In fact, we found major discrepancies between the FIGO stage attributed in CV based on CT, and the FIGO stage attributed in IPOLFG based on MRI, of which the case presented in Figure 8 is a paramount example.

Our results indicate that the majority of patients were either staged solely based on clinical findings or with the adjunct help of ultrasound and/or radiographic exams. The limitations of such staging are well known: clinical-based staging is highly inaccurate [39, 40], transrectal and/or transvaginal ultrasound do not accurately evaluate lymph node involvement [42, 43] and radiographic exams have significantly lower accuracy than CT [42].

One could also argue that the discrepancies found are due to the natural progression of untreated disease. For the 46 women who did not receive any kind of treatment in CV, the median time between the diagnosis of CC and the beginning of treatments in IPOLFG was of 6 months. It has been shown that treatment delays of only 3 months are associated with increased risk of tumour growth, lymph node metastasis and development of cancer-related complications [44]. Advanced stage tumours have particularly rapid proliferation rates [45] and, in those cases, the delay between diagnosis and treatment beginning may be sufficient to allow significant disease progression and consequent alterations in FIGO staging. A combination of all the above-mentioned factors may justify the significantly higher proportion of cases of advanced stage disease (53.5%) encountered in IPOLFG. Our results are in line with published literature, which reveal that up to 80% of all cases of CC in LMICs are diagnosed in an advanced stage [46].

### Treatment strategies and follow-up

Regarding treatment, all patients had their therapeutic strategy established in a multidisciplinary setting upon arrival to IPOLFG, according to the state-of-the-art guidelines [13, 18]. It should be noted that 20.6% had already received some kind of therapy in CV – 3.4% were surgically treated and 17.2% had started chemotherapy in CV. In recent years, there has been much debate regarding the role of neoadjuvant chemotherapy (NACT) in the treatment of CC [47–51]. Even though our sample is too small to allow us to draw any major conclusions regarding the role of NACT, we did not observe significant differences in the OS rate between the patients who started chemotherapy in CV and the ones who only started chemotherapy in IPOLFG. Our results are in line with a recent multinational multicentre study, whose preliminary results reveal similar 5-year OS rates for patients treated with NACT and surgery versus patients treated with concomitant chemoradiation [51].

In our cohort, 69% of patients (*n* = 40) were considered to be successfully treated in IPOLFG, did not develop persistence, recurrence or metastatic spread and were disease-free at the end of follow-up. Among those, 77.5% (*n* = 31) chose to return to CV soon after finishing treatments, and it was not possible to retrieve any further information regarding their clinical status from there on. The remaining 22.5% (*n* = 9) are still being followed in IPOLFG.

It is known that the great majority of CC recurrences occur in the first 2 to 3 years after the initial diagnosis [7, 11, 52, 53], and some studies have shown that disease recurrence can occur as early as 6 months after treatment initiation [54]. Late recurrences, occurring 5 or more years after diagnosis, are uncommon in CC [7, 55]. It should be noticed that disease-free patients who chose to return to CV upon treatment completion, were only followed for a median time of 6 months in IPOLFG. In this subset of women, a few cases of cancer recurrence or late disease complications may have been missed. Nonetheless, if cancer-related complications occurred in CV, those women could have once again been referred to IPOLFG, and no cases of re-referral of previously treated patients were observed. Unfortunately, it is not possible to know for sure if late disease-related complications did not occur among those patients. Current protocols do not comprise shared follow-up strategies, but their establishment would certainly be favourable, so that the long-term outcomes of patients can be accurately assessed.

The remaining 31% of women developed cancer-related complications, including disease persistence (17.2%), recurrence (3.4%), metastatic spread (12.1%) and/or cancer-related death (10.3%). The overall mortality among our cohort is similar to the one reported in CV – 9.75% [3] – highlighting the need to ease the referral of patients to Portuguese hospitals, so that the overall prognosis of women can be even greatly improved.

The median OS in our cohort was of 73.2 months, and the OS was significantly different between FIGO stages, decreasing as the FIGO stage increased. The notable is exception concerns FIGO stage II disease, but since only one death occurred among the patients diagnosed with this disease stage, this represents an undersampling bias. We also observed that the median OS was much longer in patients included in the second subgroup versus patients included in the third subgroup, which was also expected since patients included in the third subgroup are the ones who developed cancer-related complications.

Although the majority of patients presented in IPOLFG with advanced-stage disease, the survival rate in our sample was of 89.7%. Our results are favourable compared to the reality of many other African countries, where the relative survival from CC is estimated to be of 69.8%, 44.5% and 33.1% for 1, 3 and 5 years after diagnosis, respectively [56]. However, there is still a long road ahead in order to achieve the WHO Cervical Cancer Elimination Strategy Targets for 2030, which includes a 90% rate of HPV vaccination by the age of 15, a 70% screening rate at 35 and 45 years of age and a 90% rate of treatment among women diagnosed CC, in SSA countries in general, and CV, in particular [57].

### Limitations

The authors acknowledge that this study has some limitations. On the one hand, it is a retrospective study, with all the potentially inherent biases. Nonetheless, it should be noted that IPOLFG works with an extensive clinical registration system that presents accurate recordkeeping, allowing the identification of all potentially electable patients. On the other hand, the authors acknowledge that post-treatment follow-up was short, limiting the evaluation of 5-year survival or disease-free survival rates. The creation of shared follow-up protocols between Portugal and CV could certainly improve this setting.

## Conclusions

Our study highlights that there is still a huge disparity in the availability of diagnostic exams and treatment options for CC between Portugal and CV, which certainly impair the prognosis of women diagnosed with CC in CV. However, through the health cooperation agreement established between Portugal and CV, these women gain access to state-of-the-art diagnosis and treatment that clearly has a positive impact on the disease outcome. We were able to observe that the OS for the women included in our study was significantly better than what has been reported in many other SSA countries.

Our findings endorse the usefulness of health cooperation agreements between high and low-income countries, and can be seen as a model that could be applied for other African countries, in order to improve the global diagnostic and treatment response to CC.

## Conflicts of interest

The authors declare that they have no conflicts of interest.

## Funding

The authors declare that they have not received any kind of funding for the development of this work.

## Authors’ contributions

All authors reviewed and revised the paper critically for important intellectual content, gave final approval of the version to be published and are accountable for all aspects of the work. AF and TMC were responsible for the study design. ATV and RC collected the data. ATV and RC performed the statistical analysis. ATV was responsible for manuscript writing and editing.

## Figures and Tables

**Table 1.** Characterisation of relevant demographic characteristics and risk factors. Variables were summarised as medians and interquartile ranges.

Total numbers of patients (*n* =58)	Median	Range
Diagnosis (age)	56.0	49.8 – 61.0
Menarche (age)	13.5	12.0 – 14.3
Menopause (age)	48.0	42.5 – 50
No of pregnancies	6.5	4.8 – 9.5
No of deliveries	6.5	3.8 – 8.9
AFSI	15.5	14.8 – 18.8
No of sexual partners	1.5	1.0 – 3.0

**Table 2.** Characterisation of relevant risk factors. Categorical variables are presented as frequencies and corresponding percentages of positive cases for each risk factor.

Risk factors	No	Percentage
Tobacco smokers (active)	2	3.4%
Tobacco smokers (ex-smoker)	1	1.7%
Family history of cancer	2	3.4%
Immunosuppression	17	29.3%
HIV+	2	3.4%
HBV+	14	24.1%
HCV	1	1.7%
HPV DNA+	25	43.1%

**Table 3.** HPV genotype profile of our sample. We present the HPV genotyping profile of our sample in descending order of frequency.

HPV genotypes	Number of patients
HPV 16	18
HPV 33	2
HPV 53	2
HPV 68	2
HPV 18	1
HPV 19	1
HPV 25	1
HPV 31	1
HPV 35	1
HPV 39	1
HPV 44	1
HPV 45	1
HPV 56	1
HPV 63	1
HPV 70	1
HPV 73	1

**Table 4.** FIGO staging of the CC in CV and in IPOLFG. The absolute number and corresponding percentage of cases within each FIGO stage and substage are presented.

FIGO stage – CV	No	Percentage	FIGO stage – IPOLFG	No	Percentage
**Stage I**	**16**	**27.6%**	**Stage I**	**11**	**19%**
IA1	0		IA1	1	
IA2	0		IA2	0	
IB1	8		IB1	3	
IB2	5		IB2	4	
IB3	2		IB3	3	
Stage I, not specified	1		Stage I, not specified	0	
**Stage II**	**22**	**37.9%**	**Stage II**	**16**	**27.6%**
IIA1	0		IIA1	2	
IIA2	1		IIA2	0	
IIA (not specified)	1		IIA (not specified)	0	
IIB	20		IIB	14	
**Stage III**	**9**	**15.5%**	**Stage III**	**24**	**41.4%**
IIIA	0		IIIA	0	
IIIB	7		IIIB	0	
IIIC1	2		IIIC1	18	
IIIC2	0		IIIC2	6	
**Stage IV**	**0**	**0%**	**Stage IV**	**7**	**12%**
IVA	0		IVA	3	
IVB	0		IVB	4	
Stage not available	11	19%	Stage not available	0	0%
